# Removal of an over-the-scope clip using balloon dilation

**DOI:** 10.1055/a-2757-3420

**Published:** 2026-01-22

**Authors:** Thomas Lambin, Sarah Leblanc, Bertrand Napoleon, Fabien Fumex, Rodica Gincul, Vincent Lepilliez

**Affiliations:** 1 89686 Digestive Endoscopy Unit, Hôpital Privé Jean Mermoz, Lyon, France

Over-the-scope (OTS) clips have revolutionized the management of gastrointestinal perforations, ulcers, and fistulas 1 . In some cases, removing these clips is necessary, but due to their strong grip, typical forceps often fail. There is a dedicated device for removal, but it can be difficult to obtain. Various alternative techniques have been described, such as plasma argon coagulation, endoscopic submucosal dissection (ESD) or yttrium aluminum garnet crystal (YAG) laser 2 . Here we describe the case of an OTS clip removed using hydrostatic balloon dilation.

This case involves a 71-year-old patient who underwent a cystectomy and prostatectomy and subsequently developed a fistula between the neobladder and the rectum. Initially, this fistula was treated by ESD followed by the placement of an OTS clip. Due to the persistence of a fistulous tract, it was decided to remove the OTS clip with balloon dilation as a new treatment ( Video 1 ). The macro-clip was located at the anterior wall of the rectum ( Fig. 1 ). A 15-mm diameter hydrostatic balloon was used, which was inserted within the loops of the OTS clip using a guidewire ( Fig. 2 ). Once the balloon was properly positioned, it was dilated within the macro-clip up to 15 millimeters ( Fig. 3 ). Dilation of the macro-clip reopened its jaws, which subsequently closed on and pierced the ballon. The dilation balloon was then removed, with the OTS clip attached to the ballon. Endoscopic control showed no local complications. The successful removal of the macro-clip allowed a new ESD treatment of the fistulous tract and the successful placement of a new macro-clip, efficiently closing the fistula.

Removal of an over-the-scope clip using balloon dilation.Video 1

**Fig. 1:**
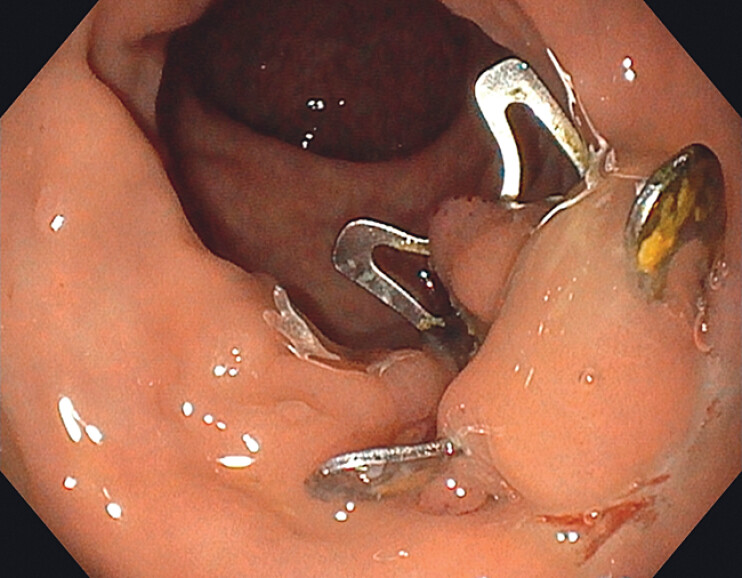
The over-the-scope (OTS) clip located at the anterior wall of the rectum.

**Fig. 2:**
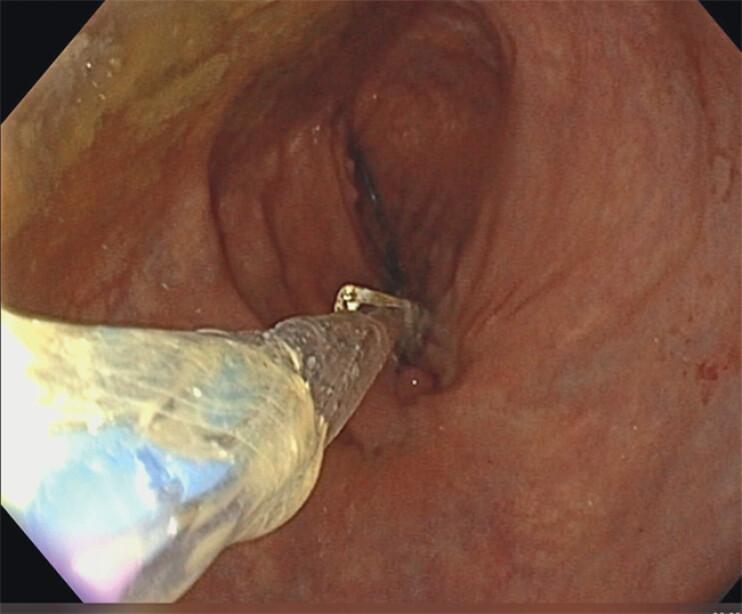
A hydrostatic balloon was inserted within the loops of the OTS clip.

**Fig. 3:**
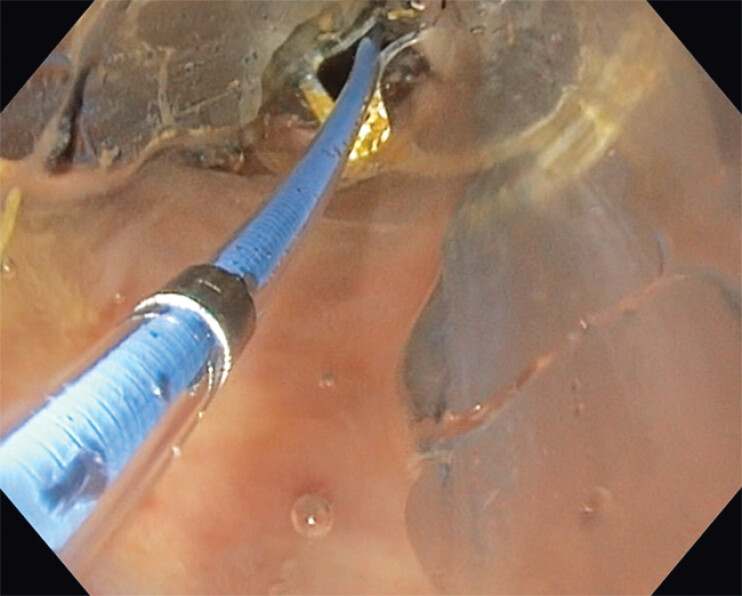
Dilation of the balloon.

This case illustrates the use of a standard hydrostatic dilation balloon for removing a macro-clip, which constitutes an interesting alternative to other previously described methods.

Endoscopy_UCTN_Code_TTT_1AQ_2AH

Citation Format

Endoscopy 2025; 57: E999–E1000. DOI: 10.1055/a-2651-0084

## Notes

Endoscopy E-Videos https://eref.thieme.de/e-videos
*E-Videos* is an open access online section of the journal *Endoscopy* , reporting on interesting cases and new techniques in gastroenterological endoscopy. All papers include a high-quality video and are published with a Creative Commons CC-BY license. Endoscopy E-Videos qualify for HINARI discounts and waivers and eligibility is automatically checked during the submission process. We grant 100% waivers to articles whose corresponding authors are based in Group A countries and 50% waivers to those who are based in Group B countries as classified by Research4Life (see: https://www.research4life.org/access/eligibility/ ). This section has its own submission website at https://mc.manuscriptcentral.com/e-videos .
